# Cardiorespiratory fitness and sports activities in children and adolescents with solitary functioning kidney

**DOI:** 10.1186/s13052-016-0255-6

**Published:** 2016-04-27

**Authors:** Giancarlo Tancredi, Caterina Lambiase, Alessandra Favoriti, Francesca Ricupito, Sara Paoli, Marzia Duse, Giovanna De Castro, Anna Maria Zicari, Giovanna Vitaliti, Raffaele Falsaperla, Riccardo Lubrano

**Affiliations:** Pediatric Department, “Sapienza” University of Rome, Viale Regina Margherita 324, Rome, 00161 Italy; General Pediatrics Operative Unit, Vittorio-Emanuele University Hospital, University of Catania, via S. Sofia, Catania, 78-95123 Italy

**Keywords:** Solitary functioning kidney, Physical activity, Cardiorespiratory fitness, Lung function tests

## Abstract

**Background:**

An increasing number of children with chronic disease require a complete medical examination to be able to practice physical activity. Particularly children with solitary functioning kidney (SFK) need an accurate functional evaluation to perform sports activities safely. The aim of our study was to evaluate the influence of regular physical activity on the cardiorespiratory function of children with solitary functioning kidney.

**Method:**

Twenty-nine patients with congenital SFK, mean age 13.9 ± 5.0 years, and 36 controls (C), mean age 13.8 ± 3.7 years, underwent a cardiorespiratory assessment with spirometry and maximal cardiopulmonary exercise testing. All subjects were divided in two groups: sedentary (S) and trained (T) patients, by means of a standardized questionnaire about their weekly physical activity.

**Results:**

We found that mean values of maximal oxygen consumption (VO_2_max) and exercise time (ET) were higher in T subjects than in S subjects. Particularly SFK-T presented mean values of VO_2_max similar to C-T and significantly higher than C-S (SFK-T: 44.7 ± 6.3 vs C-S: 37.8 ± 3.7 ml/min/kg; *p* < 0.0008). We also found significantly higher mean values of ET (minutes) in minutes in SFK-T than C-S subjects (SFK-T: 12.9 ± 1.6 vs C-S: 10.8 ± 2.5 min; *p* <0.02).

**Conclusion:**

Our study showed that regular moderate/high level of physical activity improve aerobic capacity (VO_2_max) and exercise tolerance in congenital SFK patients without increasing the risks for cardiovascular accidents and accordingly sports activities should be strongly encouraged in SFK patients to maximize health benefits.

## Background

Solitary functioning kidney (SFK) is a congenital or acquired condition that can be associated with chronic kidney disease (CKD) and/or with end-stage renal disease [1]. Congenital SFK is due to unilateral renal agenesis, to renal aplasia or to multicystic dysplastic kidney [2]. The most frequent causes of nephrectomy in pediatric age are: Wilms tumor, renal trauma and hydronephrosis [3]. SFK may be an isolated congenital malformation or may be associated with others anomalies, such as skeletal, gastrointestinal, cardiac and respiratory abnormalities [4].

Literature data have shown that SFK patients usually develop a compensatory hypertrophy, which is characterized by a concomitant compensatory glomerular filtration. Brenner et al. performed a study on animal models, supposing that the compensatory hypertrophy in SFK patients is linked to processes of hyperfiltration in residual nephrons [5]. This determines the onset of proteinuria and arterial hypertension, leading to glomerular sclerosis and end stage renal disease (ESRD) [6].

Actually, with regards to SFK patients, different opinions have been expressed: some authors stated that the SFK clinical evolution does not differ from that of healthy subjects while others observed an increased risk of kidney progressive damage [6, 7].

It is well known that physical activity represents one of the major components to improve quality of life and to reduce the mortality risk from cardiovascular disease and other causes (cancer, obesity, diabetes) [8]. However, in children with CKD the risks and benefits of a regular physical activity are still not well established. Particularly in children who underwent a renal transplantation, the practice of sport activities for more than three hours/week increases cardiorespiratory performance [9]. Other authors found that the maximal aerobic capacity (VO_2_max) decreases in the early stages of CKD in children [10].

The aim of our observational case–control study is to assess the influence of regular physical activity on the cardiorespiratory function of children with SFK, in order to promote a safe sport activity in this group of subjects.

## Methods

### Study population

We included 29 patients (22 males; 7 females) affected by congenital SFK, with a mean age of 13.9 ± 5.0 years (range 6 to 18). They were compared with a group of age matched 36 healthy subjects (25 males; 11 females), with mean age 13.8 ± 3.7 years (range 6 to 18). The minimum sample size of patients was determined based on power analysis for sample-size calculation. A preliminary medical examination was performed on each patient including clinical history. Standing height was measured using a standard stadiometer to nearest 0.1 cm and body weight was measured to the nearest 0.1 kg on a mechanical scale. All subjects were consecutively enrolled from the Pediatric Department of “Sapienza”, University of Rome from September 2014 to June 2015.

### Inclusion criteria

All participants included in this study had the following characteristics:

- normal values of Glomerular Filtration Rate;
- free from any pharmacological treatment;
- blood pressure below the 90^th^ centile on ambulatory blood pressure monitoring evaluated according to the update of the American Heart Association on ambulatory blood pressure monitoring in children and adolescents [11, 12];
- absence of contraindications to perform a clinical exercise testing according to ATS/ACCP statement 2003 [13];
- absences of physical or cognitive impairments;

None of the participants were excluded from the protocol study.

### International Physical Activity Questionnaire (IPAQ)

A standardized questionnaire (IPAQ) was administered to all subjects and their parents to investigate the time dedicated to weekly physical activities. IPAQ assesses physical activity undertaken across a comprehensive set of domains including leisure time, home activities, school-related and transport-related activity. Participants were instructed to refer to all domains of physical activity. The specific types of activity that are assessed are walking, moderate-intensity activities and vigorous intensity activities; frequency (measured in days per week) and duration (time per day) are collected separately for each specific type of activity. According to IPAQ questionnaire, three levels of physical activity have been determined as follows:

- High level of physical activity: vigorous-intensity activity on at least 3 days achieving a minimum total physical activity of at least 1500 MET-minutes/week or 7 days of any combination of walking, moderate-intensity or vigorous-intensity activities achieving a minimum total physical activity of at least 3000 MET-minutes/week.
- Moderate level of physical activity: 3 or more days of vigorous-intensity activity of at least 20 min per day or 5 or more days of moderate-intensity activity and/or walking of at least 30 min per day or 5 or more days of any combination of walking, moderate-intensity or vigorous-intensity activities achieving a minimum total physical activity of at least 600 MET-minutes/week.
- Low level of physical activity: who not meet criteria for high and moderate category.

Particularly, MET represents an index that accurately defines the physical activity, considering that its value at rest is approximately 3.5 ml O_2_/kg/min. The selected MET levels were obtained from the 2000 “Compendium of physical activities: an update of activity codes and MET intensities” to include moderate-intensity activities between 3 and 6 METs (e.g.: jogging, walking the dog, tennis) and a vigorous-intensity activities as > 6 METs (e.g.: bicycling, roller blading, volleyball) [14].

In the present study, we defined trained (T) those subjects with moderate and high physical activity and sedentary (S) patients with low level of physical activity. Therefore we attained four groups: 11 patients with SFK (SFK-S) and 12 healthy subjects (C-S) practicing low level of physical activity, 18 patients with SFK (SFK-T) and 24 healthy subjects (C-T) practicing moderate to high level of physical activity. Table 1 shows age and anthropometric parameters and blood pressure of all included subjects.

**Table 1:** Age, anthropometric parameters and blood pressure in all patients, Trained (T) and Sedentary (S), with solitary functioning kidney (SFK) and in controls (C)

	SFK-S	C-S	SFK-T	C-T	*P* value
	*n* = 11	*n* = 12	*n* = 18	*n* = 24	
Age (yrs)	14.2 ± 4.4 (6.1–18.0)	14.3 ± 3.3 (6.3–18.0)	13.7 ± 5.4 (6.2–18.0)	13.5 ± 3.8 (6.0–17.8)	ns
Height (cm)	160.9 ± 26.4 (113.0–183.0)	163.1 ± 16.3 (117.0–180.0)	158.6 ± 29.0 (116.0–182.0)	162.5 ± 5.9 (119.0–178.0)	ns
Weight (kg)	60.2 ± 26.4 (25.0–84.0)	59.1 ± 14.7 (28.0–75.0)	57.7 ± 26.7 (26.0–72.0)	57.3 ± 7.2 (31.0–74.0)	ns
LBM (kg)	48.4 ± 21.1 (20.1–65.2)	48.3 ± 11.1 (22.2–60.4)	46.6 ± 21.5 (22.3–60.1)	47.7 ± 5.5 (25.1–59.2)	ns
BMI (kg/m^2^)	21.6 ± 4.8 (14.1–30.4)	21.7 ± 2.2 (16.5–24.2)	21.3 ± 4.3 (14.2–31.7)	20.2 ± 2.0 (17.1–24.4)	ns
BMI - z Score	0.17 1.25 (−2.2–1.2)	0.20 0.69 (− 1.4– 0.9)	0.06 1.22 (−1.8 – 2.1)	−0.22 0.86 (−1.4 – 1.0)	ns
SBP (mmHg)	113.6 ± 17.5 (85.0–130.0)	114.9 ± 11.2 (95.0–130.0)	116.7 ± 16.5 (85.0–130.0)	112.6 ± 6.1 (100.0–125.0)	ns
DBP (mmHg)	60.9 ± 12.8 (60.0–80.0)	66.7 ± 6.0 (50.0–75.0)	69.4 ± 9.4 (50.0–80.0)	67.8 ± 4.1 (59.0–71.0)	ns

*BMI* Body Mass Index, BMI z- score, *LBM* Lean Body mass, *SBP* Systolic Blood Pressure, *DBP* Diastolic Blood Pressure, *ns* not statistically significant. Values are expressed as mean ± standard deviation (range)

### Spirometry and cardiopulmonary exercise testing

Spirometry was performed in accordance to the European Respiratory and American Thoracic Society (ERS-ATS) statements using a commercial device Cosmed Quark PFT (Rome, Italy) to determine the following parameters: forced vital capacity (FVC), forced expiratory volume in the first second (FEV_1_), ratio between the FEV_1_ and FVC, peak expiratory flow (PEF) and forced expiratory flow at 50 % of vital capacity (FEF_50_). All parameters have been expressed as percentage of the normal values compared to the predicted reference equations obtained by algorithms according to age, gender, height and weight [15].

Each patient underwent a 12-lead electrocardiogram (PC-ECG 1200 wireless - Norav Medical Ltd, software version 5.515, Israel) with the subject on supine and standing position, in order to have the standard electrocardiogram tracing before the exercise test.

All patients performed a maximal incremental exercise test on treadmill, in accordance with Bruce protocol, consisting of sequential increase in speed and slope every 3 min until exhaustion (breathlessness and leg muscle pain) and/or a hearth rate ≥ 85 % of maximum (HRmax) calculated with the formula 220 – age in years [13].

Systolic blood pressure (SBP) and diastolic blood pressure (DBP) were measured in all subjects at rest and every 3 min during the exercise test by the same physician. Before each test calibration of the gas analyses and flow transducers were performed. During the test, all subjects were connected by face mask to a breath-by-breath analyzer to measure the following parameters: respiratory frequency (RF), minute ventilation (VE), tidal volume (VT), oxygen consumption (VO_2_ml/min/kg), and exercise tolerance expressed in minutes (ET) [16]. VO_2_max was also corrected for estimated lean body mass (LBM) using the following equations: male: LBM: 1.10 × weight – [128 × (weight^2^/height^2^)]; Female: LBM : 1.01 × weight – [148 × (weight^2^/height^2^)], [17].

### Kidney function

The glomerular filtration rate (GFR) for each child in the SFK group was calculated as creatinine clearance (CrCl) on a 24-h urine collection, according to the protocol previously described [18].

### Statistical analysis

All statistical calculations were performed using SPSS for Windows version 18.0 (SPSS, Inc., Chicago, Illinois, USA). Height, weight, body mass index (BMI), SBP and DBP have been expressed as mean ± standard deviation (Mean ± SD) and range.

Height, weight, BMI values were further calculated as z scores from healthy children (z score is the number of Standard Deviations that a value deviates from the expected mean value of healthy subjects), using the Centers for Disease Control and Prevention growth charts 2000 [19].

The significance of differences between the two study groups was tested using Student’s two-tailed unpaired *t*-test and, for each subgroup, statistical differences were analyzed by an analysis of variance (ANOVA). A *p*-value ≤ 0.05 was considered statistically significant.

### Ethics, consent and permissions

Informed consent was obtained from the patients’ caregivers and our research protocol was approved by the scientific ethics committee of the “Sapienza” University of Rome (Rif. 3399).

## Results

Patients with SFK showed mean values of anthropometric parameters and blood pressure similar to healthy controls, independently from the level of physical activity (Table 1).

Glomerular filtration rate, expressed in mL/min/1.73 m^2^, was normal in all subjects, without differences between groups: SFK-T = 100.35 ± 34.56, SFK-S = 103.46 ± 43.22, controls (T and S) = 106.77 ± 44.12.

ECG analysis at rest showed in one patient (3.4) with SFK and in two controls (5.5 %) isolated premature supraventricular complexes, which disappeared during exercise reflecting the normal prevalence of arrhythmias in the pediatric population [20].

Spirometric indexes in all groups, expressed as percentage of predicted values, were similar and within the normal limits (Table 2).

**Table 2:** Spirometric parameters in all subjects, Trained (T) and Sedentary (S) with solitary functioning kidney (SFK) and in controls (C)

	SFK S	C S	SFK T	C T	*P*<
	*n* = 11	*n* = 12	*n* = 18	*n* = 24	
FEV_1_ (l)	102.9 ± 9.3 (80.8–116.4)	96.9 ± 8.9 (81.0–112.6)	97.5 ± 19.6 (79.9–121.4)	102.8 ± 4.7 (97.9–114.0)	ns
FVC (l)	97.4 ± 8.6 (81.3–109.5)	97.3 ± 9.1 (84.3–116.0)	94.8 ± 19.3 (80.7–121.9)	102.3 ± 5.8 (92.8–116.0)	ns
FEV_1_/FVC (%)	89.8 ± 3.8 (83.5–94.7)	93.5 ± 25.4 (89.7–110.5)	89.3 ± 5.4 (73.3–94.6)	89.9 ± 1.6 (87.0–92.4)	ns
FEF_50_ (l/s)	97.0 ± 14.4 (68.9–124.3)	93.2 ± 15.0 (71.0–114.0)	104.3 ± 28.1 (69.6–154.0)	102.8 ± 5.9 (92.9–117.0)	ns

*FEV*_*1*_ Forced Expiratory volume in the first second, *FVC* forced vital capacity, *ns* not statistically significant. Values are expressed as mean ± standard deviation (range)

The mean values of the cardiopulmonary parameters measured at maximum workload in all subjects are listed in Table 3. We found that VE, RF, VT and HRmax were similar and within the normal range independently from the level of physical activity in SFK as well as controls. The values of systemic blood pressure measured during the exercise test were normal without significantly differences in all groups.

**Table 3:** Cardiorespiratory and metabolic parameters measured at the maximum workload in all subjects

	SFK-S	C-S	SFK-T	C-T	SFK-S vs C-S	SFK-T vs C-S
	*n* = 11	*n* = 12	*n* = 18	*n* = 24	*P*=	*P*=
VE (l/min)	67.7 ± 20.3 (27.9–90.0)	71.0 ± 16.6 (37.4–97.0)	69.8 ± 21.9 (39.7–95.0)	75.1 ± 13.3 (37.8–86.6)	ns	ns
VE (ml/kg/min)	1348.7 ± 722.3 (416–2820)	1320.1 ± 185.6 (1036–1672)	1393.4 ± 595.3 (571–2650)	1475.9 ± 245.0 (1065–1939)	ns	ns
RF (breath/min)	50.2 ± 9.4 (32.0–58.0)	53.6 ± 7.3 (43.1–64.3)	50.4 ± 12.0 (31.0–75.0)	53.1 ± 6.8 (40.4–68.0)	ns	ns
VT (l)	1.5 ± 0.5 (0.6–2.2)	1.3 ± 0.3 (0.8–1.8)	1.5 ± 0.7 (0.5–2.5)	1.2 ± 0.2 (0.7–1.6)	ns	ns
VT (ml/kg/min)	28.4 ± 8.8 (17.7–42.5)	27.5 ± 5.1 (15.5–31.8)	28.0 ± 8.5 (14.5–41.1)	28.0 ± 4.1 (20.5–34.8)	ns	ns
VO_2_max (ml/Kg/min)	39.9 ± 5.7 (27.9–60.2)	37.8 ± 3.7 (33.9–48.7)	44.7 ± 6.3 (28.5–58.4)	44.3 ± 3.7 (38.7–50.5)	ns	<0.0008
VO2 max / LBM (ml/Kg/min)	49.6 ± 7.1 (34.8–70.4)	46.2 ± 5.3 (39.9–60.9)	55.3 ± 9.6 (37.3–70.1)	53.2 ± 5.4 (43.8–65.3)	ns	<0.002
HRmax (beat/min)	189.3 ± 6.1 (177–196)	192.7 ± 9.6 (176–210)	190.2 ± 11.9 (171–206)	197.0 ± 6.8 (178–210)	ns	ns
SBPmax (mmHg)	140.0 ± 24.8 (110–155)	156.5 ± 13.8 (120–175)	145.6 ± 22.6 (110–170)	149.0 ± 7.9 (125–160)	ns	ns
DBPmax (mmHg)	65.0 ± 9.7 (50–80)	69.1 ± 0.5 (50–72)	72.5 ± 8.3 (60–75)	69.0 ± 5.1 (60–79)	ns	ns
Exercise time (min)	10.8 ± 2.5 (9.0–12.0)	11.5 ± 1.5 (10.5–12.5)	12.9 ± 1.6 (10.0–14.0)	13.7 ± 1.3 (11.5–15.0)	ns	<0.02

*VE* minute ventilation, *RF* respiratory frequency, *VT* respiratory frequency, *VO2max* maximal oxygen uptake, *VO2 max* / *LBM* maximal oxygen uptake/ lean body mass, *HRmax* maximal heart rate reached during exercise test, *SPBmax* maximal systolic blood pressure reached during exercise test, *DBPmax* maximal diastolic blood pressure reached during exercise test

Conversely VO_2_max was statistically significantly higher in SFK-T with respect to C-S (*p* < 0.0008), but comparable with C-T. Similarly, we obtained the same results when VO_2_max was adjusted to estimated LBM (SKT-S vs C-S: *p* < 0.002). Furthermore exercise tolerance, expressed as exercise time in minutes, was statistically significantly higher in SFK-T than C-S (*p* < 0.02).

The trained SFK population of our study practiced the following sports: swimming (8 subjects), dance (5 subjects), tennis (2 subjects), volleyball (1 subject), aerobic activities in gym (2 subject) while in controls were: swimming (6 subjects), soccer (11 subjects), volleyball (4 subject), dance (3 subject).

Moreover we observed that in our study population the percentage of sedentary subjects were higher in patients with SFK than healthy controls (SFK = 37.9 and C = 33.3 %), even if the result was not statistically significant.

## Discussion

The present study, although based on a limited number of subjects, suggests that trained subjects showed a better cardiorespiratory fitness than sedentary subjects. In particular SFK-T had significantly higher mean values of aerobic capacity (VO_2_max) and exercise tolerance (ET) than sedentary.

As we expected, the respiratory pattern (VE, RF, VT), measured at maximal workload, was in the normal range and showed similar mean values in all groups independently from their level of physical activity. Accordingly respiratory function parameters at rest (FEV_1_, FVC, FEV_1_/FVC, FEF_50_) were normal in patients with SFK and comparable to healthy subjects, showing that these patients did not present a pulmonary impairment. Since 1982, the exercise test together with spirometry and electrocardiogram before and after exertion has been mandatory in Italy for all persons wishing to engage to competitive sport [21].

To our knowledge, there are no data evaluating the pulmonary function and maximal aerobic capacity in children with SFK in relation to physical activity using a maximum exercise test performed on treadmill.

Cardiopulmonary exercise testing is essential to provide a global assessment of integrative exercise responses involving the pulmonary, cardiovascular, and skeletal muscle systems. Moreover cardiopulmonary response to exercise can also provide important information on physical efficiency as reported for other diseases [9, 22].

Patients with SFK are affected by a chronic condition in which the only functioning kidney should compensate the other, keeping global glomerular filtration within the limits of normal. To accomplish this task, the functioning kidney, over time, undergoes a process of hypertrophy and hyperfiltration, which can lead to an increase in systemic blood pressure and other possible cardiorespiratory effects [23, 24]. Consequently, it is necessary to perform a complete functional assessment in these subjects to establish an early diagnosis, to prevent further complications and suggest the most suitable sports activities. Frequently, in patients with chronic diseases, an overprotective attitude by parents has been observed, which results in a reduced physical activity of these children and, therefore, a lower exercise tolerance and an increased risk of obesity.

According to the World Health Organization guidelines all children should be physically daily active as part of play, games, sports, physical education in the context of family, school, and community activities [25, 26]. In particular, as recently demonstrated by Moore et al., playing regular exercise makes an opportunity for socialization, reduces mortality and increases life expectancy; moreover, for children and adolescents, sport participation is associated with significant mental, social and physical benefits and is able to reduce blood pressure and cardiovascular risk in patients with chronic kidney disease [25, 27]. For these reasons it is very important to promote physical activity and to know the type and the level of physical activities in these patients.

In our study we used the IPAQ questionnaire to determine the amount of physical activity in MET because it has been demonstrated as a reasonable and valid method against the accelerometer criterion [28]. Other authors with the HELENA-study, showed that in adults the IPAQ presents modest comparability with the accelerometer data for assessing physical activity in each intensity level [29].

A limitation of our study is due to the restricted number of subjects, because congenital SFK is rare. Consequently the study group is not homogeneous for gender (22 males and 7 females) and age (range 6–18 years). Moreover it could be interesting to evaluate a population study with acquired SFK, because they present a worse renal functional adaptation than congenital SFK during a long period of follow-up [30].

At present specific guidelines suggesting the more suitable sport activity for a child with SFK are not available and, in regards there are conflicting opinions. Some authors believe that subjects with solitary kidney can play any sports, accounting for a low probability of kidney injury from sports [31]. Conversely, many pediatric nephrologists agree with the American Academy of Pediatrics that, since 1994, recommended that subjects with SFK should avoid contact and collision sports [32]. In particular, most high-grade renal injuries result from motor vehicle collisions associated with numerous concomitant injuries. Sports-related blunt renal injury tends to have a different mechanism as a solitary blow to the flank [33].

Therefore, it is important to standardize the medical knowledge possibly drafting specific recommendations or guidelines to promote physical activities and sports in patients with SFK, except for contact and collision sports (martial arts, hockey, soccer, rugby, football, skiing, sledding, cycling), which could damage the solitary kidney [34, 35].

Concerning non-competitive sports nephrologists attitude is mostly positive, with the exception of severe CKD and kidney transplant recipients [36].

In accordance with Matthew, we suggest that SFK patients require, for sports participation, to undergo a preliminary medical examination including recent imaging confirming normal position and anatomy of the single kidney and without evidence of renal insufficiency, hypertension, or proteinuria. Should any of these parameters be found abnormal, consultation with a specialist for management and further decision-making is appropriate [37].

## Conclusions

The present study shows that regular moderate/high level of physical activity improves cardiorespiratory fitness in congenital SFK patients without increasing the risks for cardiovascular accidents.

We suggest that SFK patients need to be more motivated and encouraged to take part in various physical activities, except for collision and contact sports, to increase their fitness levels in order to reduce the consequence of sedentary lifestyle.

## Abbrevations

BMI: body mass index
C: control group
CKD: chronic kidney disease
CrCl: creatinine clearance
DBP: diastolic blood pressure
ET: exercise time expressed in minutes
FEF_50_: expiratory flow at 50 % of vital capacity
FEV_1_: forced expiratory volume in the first second
FVC: forced vital capacity
GFR: glomerular filtration rate
HR: heart rate
HRmax: the highest heart rate reached during a maximal exercise test
IPAQ: International Physical Activity Questionnaire
LBM: lean body mass
MET: metabolic equivalent
PEF: peak expiratory flow
RF: respiratory frequency
S: sedentary
SBP: systolic blood pressure
SFK: solitary functioning kidney
T: trained
VE: minute ventilation
VO_2_ max: maximal oxygen uptake
VT: respiratory frequency
